# Towards Immunization Financing Sustainability in Africa

**Published:** 2018-07-03

**Authors:** Amos Petu

**Affiliations:** Immunization Financing Sustainability (EPI), Intercountry Support Team East and Southern Africa, World Health Organization, Harare, Zimbabwe

**Keywords:** Africa, Financing, Immunization, Vaccines

## Abstract

Immunization programme has contributed to saving many lives from avoidable deaths and bring many other benefits, including healthier children, increased school attendance, and increased productivity. In the past 10 years, immunization as a public health intervention has expanded in target as well as number of vaccines to be delivered to a broader range of people and new vaccines. Immunization is also exceptionally of good value, returning many dollars in economic benefits for every dollar invested in immunization services. Healthy individuals are more productive, earn more, save more, invest more, consume more, and work longer: which all impact to increase a nation’s GDP. Immunization is one of the most effective, and cost-effective, public health tools that contribute to this situation. Fully immunized children have better educational outcomes and, over time, make for a more productive workforce. Consequently immunization, which must be sustained indefinitely, as a long-term investment require stable, long-term financing. A start point is a plan which is translated into funding for the programme. In sustainability a detailed planning process that assures a review of the situation leading to detailed programming in terms of response to challenges and finally culminating in costing so that funding requirements are determined and mobilised cannot be overemphasized. The experience has been varied in Africa region. While governments have made significant strides to increase funding for immunization programs over the last five years, further commitment is needed to achieve full financing and national ownership of immunization programs.

Most countries have adopted the Comprehensive Multi-year Planning framework for planning and are thus able to put together their resource needs for immunization programmes. To continue to have the necessary benefits of high coverage and cover the increased investment requirements governments will need to do more to assure robust funding in a sustainable and predictable manner. The paper tells the story of importance of planning using the cMYP processes to immunization financing sustainability as a necessary condition in the trajectory towards sustainability. This article presents the experience of countries from planning to funding, drawing on the interconnectedness of adequate planning, ability to mobilise resources and thus better move towards sustainable funding. As governments pursue high level order of planning, they are in a better position to stem overdependence on Gavi and other external support for future sustainability.

## Introduction

The immunization financing portfolio of countries has gained importance to sustain coverage and introduce new vaccines and available technologies. African countries, as well as partners, have demonstrated their concern with regards to the sustainability of immunization programme financing. It has been estimated that between 2016 and 2020, Africa will require $17 billion for vaccines and delivery cost. Projections indicate that Governments is expected to provide $6 billion while the donor community will give another $6 billion leaving a gap of about $5 billion. In the same vain, African countries are expect to derive benefit equivalent of about $224 billion in direct returns and savings from vaccine preventable diseases^1^–^2^.

GAVI Alliance at inception consider sustainability to be the ability of a country to mobilize and efficiently use domestic and supplementary external resources on a reliable basis to achieve current and future target levels of immunization performance regarding access, utilization, quality, safety and equity. While such broad understanding around sustainability to get countries to own the programme^3^, in the final analysis, a more sustainable programme will be one that moves towards self-sufficiency, that will be able to overcome shocks that may occur if the dependency on external support is suddenly withdrawn as have been the case in some countries in the past. Ensuring sustainable resources for immunization is critical in sustaining the gains, and in achieving the Global Vaccine Action Plan (GVAP) targets^4^. There are varied estimates of cost of vaccination of a child amongst countries, driven by non-vaccine costs of delivering the service, training, supervision, monitoring and tracking outbreaks, addressing population demand for services or managing programs^5^. As at 2016, Angola, Ghana, Cote d’Ivore, Congo and Nigeria already have GNI per capita greater than US$1580. As more countries in Africa pass the Gavi threshold of eligibility of US$1580 per capita, more focus will shift from partner support to domestic funding. To ensure every child receives the vaccines they need as at when due and that immunization will continue in perpetuity governments need to carefully plan and adequately budget for both vaccines and the delivery costs of immunization programs to assure long term sustainability.

Comprehensive Multi-year planning is a key management tool for national immunization programmes. It helps the EPI programme to bring together in one framework, objectives of the programme, actions to lead to results and the cost, as well as the available and anticipated funding. This way potential gap in funding can be derived. The comprehensive multiyear plan focuses on all components. This include the services to be delivered, mechanism to deliver such services using statics or mobile deliver; human resource requirement, logistics and cold chain including vehicles for distribution of vaccines and supervision, programme management and not least the vaccines. In time past EPI managers have had to develop many different plans to reach many different immunization objectives and address each of the components separately. This has not led to the requisite synergy that should exist amongst the different objectives. Developing a comprehensive multiyear plan (cMYP) presents an opportunity to consolidate programme thinking into a single document that addresses global, national and sub-national immunization objectives and strategies, and that also evaluates the costs and financing of that plan. Increasingly, cMYPs have been used to strategically guide immunization programmes and mobilise domestic resources especially for countries that are not GAVI-support eligible. The attraction is that budget preparation and arguing for funding for immunization requires that concrete cost estimates be provided. The cMYP as a tool strengthens the EPI programme in deciding the appropriate level of financial investment and the management of flow of funding towards sustainability. The starting point in the process is to plan and determine the investment requirement in financial and other resources. Such planning has been done by most countries through the comprehensive multi-year planning (cMYP) process. Increasingly, cMYPs have been used to guide immunization programmes strategically and mobilise domestic resources especially for countries that are not Gavi support eligible. In this article, we present progress of countries in their effort to assure adequate funding for immunization programmes, starting with planning. We argue that planning especially through the cMYP framework, is a necessary condition in the trajectory as such, concerted efforts should be made by government and partners to resource the process and strengthen capacity.

## cMYPs and Immunization Financing Sustainability

The cMYP process was developed as a way to overcome the challenges of raising sufficient financial resources for immunization. Guidelines to provide a general overview of the cMYP process, as well as providing detail for each of the seven steps involved in the development and implementation of the cMYP was developed and recently updated. A costing tool, as well as user guide which should both be used as the first point of reference, was also developed^6^.

As a robust process, bringing together the cost of programming and possible funding to determine gaps and thus the opportunity for resource mobilization, this has also forged close collaboration between the Ministries of Finance and Health. This is an era of insufficient resources, and competition for those that are available. The budget allocation process is a political process in which lobby, evidently defense of budget proposal are necessary. At national and provincial levels, immunization programme managers must actively engage in the budget process to ensure that appropriate funds are allocated to immunization as part of the health budget. If needed, they must also seek ways to attract additional funds such as getting off budget allocation otherwise call special intervention schemes, to the programme from extra-budgetary sources, and from sources as bilateral donors and partners as well as from sources within the country including the private sector and civil society. Also, they must engage with other programme managers to explore ways of integrating elements of routine immunization with other primary health care initiatives to drive synergies in funding as well as programmatically.

From the cMYP countries derive estimates that are used for budgeting. Thus in order to review how well the funding was with immunization, efforts was made to assess utility of cMYP.

A 2012 assessment conducted in WHO Africa Region^7^ shows that out of 36 countries that responded 32 countries indicated that cMYPs are translated into plans for resource mobilization, including securing government budgets. cMYP provided an opportunity for partners in immunization to align their funding and harmonize their plans with the strategic document. The plan is recognized as a comprehensive tool for resource mobilization among partners. Outputs from the cMYP tools have been used to prepare technical reports to Gavi such as the Annual Progress Report. The cMYP has been a useful reference document for seeking Gavi support. Involvement of local expertise in the development of cMYP has strengthened the local ability to use local economic figures and carry out comprehensive planning and costing.

**Figure 1:**
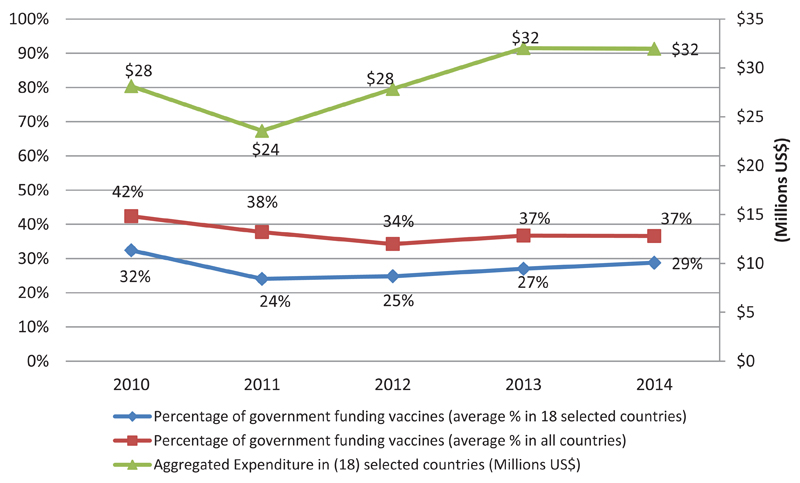
Trends in Government Expenditure on Vaccines

Since 2010, 44 out of the 46 countries in African Region of WHO reported having line items in their national budgets for purchasing vaccines. This trend in the number of countries reporting a line item for the purchase of vaccines appears relatively stable over the period. A further interrogation indicated that in a few countries, procurement of vaccines is treated as capital expenditure within government budget system. In such instances this puts constrains on the programme to draw down in cases where the Parliament is yet to pass the budget, unlike a situation if treated as recurrent expenditure items for which statutory expenditures could be made up of a percentage of the budget under consideration. The guidelines for reporting immunization expenditure indicated the cMYP as a source^8^. As such, countries that have over the years reported in the JRF immunization expenditure used a combination of sources of information including the cMYP. The various immunization financing indicators that are tracked are: Government Expenditure on Vaccines (JRF 6510); Government Expenditure on Routine Immunization (JRF 6540) to include all spending on operational aspects of service delivery such as supervisions, community mobilization and training of health workers. Also monitored is Total Expenditure on Vaccines (JRF 6520) (Figure 1) (Table 1).

From the JRF database^a^, the population weighted average was calculated for each of the indicators according to U.N. Population data on the number of live births. Government expenditures on routine immunization showed signs of an overall increase over the period 2010 to 2014, from $52 million to $58 million. Initially, the aggregated expenditure dropped from $52 million in 2010 to $49 million in 2011 after which it saw consistent increases over the remaining 3 years. The percentage of government funding for routine immunization in the region fell (on average) over the five-year period, showing a consistent decreasing trend from 50% in 2010 to 46% in 2012 after which it fell to 40% in 2013 and remained for 2014. While governments are directing more and more funds towards the routine costs in the immunization program, increasing amounts of external support, continue to diminish the overall proportion which is funded by the government. Given that routine immunization costs encompass the costs of vaccines, it seems that the decreasing trend in the percentage of government funding RI correlates with the increased introduction of new and underused vaccines supported by donors.

The percentage of reported government funding of vaccines (on average) decreased from 42% to 34% over the period 2010 to 2012, after which it climbed to 37% in 2013 and 2014. However, during this period the government expenditures on vaccines saw an overall increase overall albeit with a somewhat fluctuating trend. Initially, the government expenditure on vaccines drops from $28 million in 2010 to $24 million in 2011, before increasing by a substantial amount over the next 2 years to $32 million in 2013 where it remains stable going into 2014. 30 countries in the region showed an increase in government funding for vaccines when comparing their reported spending in 2014 with their reported figures for 2010.

Sustaining immunization coverage at high enough level to ward off insistent disease outbreaks is a costly endeavour. Thus ensuring adequate financial resources requires that fiscal space is made available to expand or maintain coverage without jeopardizing the balance in government financing solvency. Such fiscal space for immunization services is generated by both the macroeconomic and fiscal capacity of a country and by the priorities set in government budget allocations. A measure of availability of budget line for vaccine procurement as indicated in JRF is a good attempt to check the extent to which government prioritizes immunization. Broadening tax base and improving tax administration, obtaining grants, reprioritizing expenditures, improving efficiency, and, temporarily, by borrowing as means through which governments in Africa region can sort to increase funding for immunization. Economic growth creates fiscal space naturally from increased tax revenues. Within overall fiscal space for health, priority for financing immunization services requires adequate allocations to purchase vaccines and injection supplies, for vaccine delivery (cold chain equipment, management, and transport), and for immunization service delivery. But economic growth alone often is not enough to bring about increases in real government health spending.

While the government expenditure on vaccine exhibits an increasing trend, the overall decrease in the percentage of government funding vaccines comes mainly as a result of increases in total expenditure on vaccines over the period 2010 to 2014. The increasing trend in expenditures in vaccines is a result of the introduction of new and underused vaccines into AFRO country’s routine immunization schedules. The number of countries in the region which have introduced Pneumococcal Conjugate Vaccine (PCV), Rotavirus vaccine and Human Papilloma Virus (HPV) vaccine can be seen in the table below and can be seen to comply broadly with the increasing trend in vaccine expenditures. All but nine countries in the region are classified as Low or Lower-middle-income countries, which allows for Gavi eligibility in 38 countries. This high proportion of Gavi support in the region serves to explain the high uptake of PCV and Rotavirus vaccines and hence the escalating total expenditures.

Gavi has been identified^9^ as one of the few development platforms attempting to move countries towards self-sufficiency; it is unclear how well this has been true for countries in Africa. Another area that tests the financial sustainability of immunization programme in Africa is the rate at which Gavi-eligible countries default in meeting their co-financing payment as at when due. Available information (Gavi) indicates that there has been a 100% increase in the number of countries from Africa region in default from 2008 (6) to 2014 (12) with variation in trends within the years and the lowest level being 2011 (4 countries)^10^. As immunization financing review carried out in some countries, suggest that while programme financing requirement is on the increase, the sector budget ceiling and mode of budgeting that relies on historical budgeting constraints the space for additional funding for immunization. More worrisome is the fact that most of the economies had varied growth rates of between 4% and 8% within the period of 2008 and 2014. Such growth rate ordinarily signaled improved fiscal space, leading some countries to transition away from Gavi support rapidly. The Gavi support threshold of $1580 Gross Nation income (GNI) per capita, provides the benchmark above which countries will be expected to be well positioned to fund immunization from national resources. A few of these countries have featured on the co-financing default list^b^ putting a question mark on sustainability of funding going forward beyond Gavi support. The Regional Vaccine Action Plans present opportunities for collective action by countries to jointly secure the collective good of a fully, sustainably immunized African continent. The needed resilience around immunization financing sustainability could be assured with countries having supporting legislation backed by advocacy efforts that target budget appropriation at national as well as sub-national governments. The current under provisioning of resources cannot support an increasing programme demand for a series of investments that must be made for vaccine introductions to be effective and for children to be rapidly reached. Indeed a situation where some countries could meet the Gavi co-financing requirement yet unable to procure traditional vaccine using national government funds does little to support sustainability in whatever form. Lead responsibility for eventual sustainability rests with government politically acceptance of the need for self-sufficiency ranging from the domestic production of safety boxed to a long-term plan for vaccine production.

## Conclusion

The financial sustainability of immunization programmes remains a challenge in the face of a fiscal crisis, especially as countries have to introduce new vaccines. Some countries have the opportunity of funding support from Gavi. The extent to which countries has interpreted such support as substitution of government funding will always be a moot point. Furthermore, immunization has to compete with other health system priorities, in the face of dwindling revenue from government, recourse to the cost-effectiveness of immunization as a public health good should be used more in advocacy for better funding. The cMYP presents the EPI programme to go through a process that will ensure priority setting and on the basis of that argue for government budget allocation. The process establishes a matching of resource with expected cost to determine gaps. To the extent that costing and planning are fundamental, subsequent government funding based on the plans and cost will assure sustainability. Government and partners should use the cMYP process as a mechanism to establish priority for mechanism not only for new vaccine introductions but also for delivery strategies as this have implications for cost of the programme.

## Figures and Tables

**Table 1:** Trend in JRF Indicators by Years

Government Routine Immunization Expenditures:	2010	2011	2012	2013	2014
% of total RI funded by government in the region (average)	50%	48%	46%	40%	40%
% of total RI funded by government in (18) selected countries (average)	40%	35%	37%	36%	33%
Aggregated Expenditure in (18) selected countries (Millions US$)	52	49	50	52	58
Expenditure Per Live Birth in (18) selected countries (PWA US$)	5.7	5.3	5.3	5.3	5.8
Government Vaccine Expenditures as:	2010	2011	2012	2013	2014
% of total vaccines funded by government in the region (average)	42%	38%	34%	37%	37%
% of total vaccines funded by government in (18) selected countries (average)	32%	24%	25%	27%	29%
Aggregated Expenditure in (18) selected countries (Millions US$)	28	24	28	32	32
Expenditure Per Live Birth in (18) selected countries (PWA US$)	3.1	2.5	2.9	3.3	3.2

## Footnotes

a JRF database is hosted in WHO. It has reported data from countries for various years.

b Gavi co-financing information by country is accessed through http://beta.gavialliance.org
